# Identification of the Right Environmental KPIs for Manufacturing Operations: Towards a Continuous Sustainability Framework

**DOI:** 10.3390/ma15217690

**Published:** 2022-11-01

**Authors:** Ziyad Sherif, Shoaib Sarfraz, Mark Jolly, Konstantinos Salonitis

**Affiliations:** Sustainable Manufacturing Systems Centre, School of Aerospace, Transport and Manufacturing, Cranfield University, Cranfield, Bedfordshire MK43 0AL, UK

**Keywords:** sustainable manufacturing, indicators, environment, selection criteria

## Abstract

Sustainable manufacturing has grown into a major subject of discussion between individuals and organisations around the world. This is attributed to the recognition of the urgency in advancing sustainable manufacturing due to the diminishing non-renewable resources, stricter regulations related to environmental impacts and the increasing consumer preference for environmental-friendly products. However, manufacturing companies have been confronted with a decision on which KPIs to select for appraising their processes, and how they should interpret these KPIs in transforming their processes towards a sustainable future. This paper presents a structured framework for the manufacturing industries to identify the right environmental KPIs. It includes building a database for environmental KPIs, categorising, ranking, and composing a final KPI set for specified targets. The developed method allows for the selection of the most effective KPI in representing a specified target as well as identifying unmonitored environmental aspects. The framework has been corroborated by subject matter and industry experts in which the potential benefits have been verified.

## 1. Introduction

The concept of sustainability was pioneered by a report published in 1987 by the World Commission on Environment and Development (WCED). This initiated the vast research on the matter and the incorporation of this notion in all aspects of life from individuals to corporations and whole countries all over the world [1,2]. A prominent concept followed in 1997 which considered the construction of sustainability was based on three pillars [3], known as the triple bottom line (TBL), which correlates the sustainable systems to social, economic and environmental positions [4,5]. Accordingly, many organisations are under pressure from their stakeholders to identify performance in terms of TBL as a method to present a broader perspective rather than reporting in terms of a particular aspect (i.e., cost) [6,7,8]. It is worth mentioning that such pressure could be imposed internally by management by setting environmentally guided targets [9], or externally, by the environmentally conscious consumers’ demands. Thus, in order to proactively respond to these new demands, decision-makers within organisations require up-to-date and accurate performance information that represents the various aspects of their business operations [10,11,12]. However, due to the complexity of the subject, it can also rely on the level at which the performance is being assessed [13], the agility of the organisation’s structure and its elements which poses additional challenges to evaluating performance [14]. According to Yun and Yigitcanlar [15], organisations that are continuously and consciously altering their business model towards sustainable development can stay competitive. Multiple studies have addressed the sustainable performance of organisations and their willingness to innovate as key factors that act hand in hand toward continuous development [16,17]. Another major aspect of continuous development is the organisation’s pursuit of value-adding activities to maximise competitiveness [4]. Sustainable development is at the foundation of such a goal, it leads organisations to operate in ways which is less harmful to the environment [18]. This is achieved by considering their resource consumption which introduces further positive effects on both the cost and the brand image [19].

Among the organisations with the most impact, and definitely, the most impacted is the manufacturing industry. It contributed USD 14.17 trillion to the world’s GDP in 2018 and the emission of 24% of global greenhouse gases (GHG) in 2016 [20]. Therefore, sustainable manufacturing has grown into a prominent topic of discussion between business stakeholders around the world. This is attributed to the recognising of the urgency in advancing sustainable manufacturing due to the diminishing non-renewable resources, stricter regulations related to environment and occupational health and safety and increasing consumer preference for environmentally friendly products [21].

It is vital to first define sustainable manufacturing which is the creation of products that diminish negative environmental impacts while conserving energy and natural resources using economically sound processes that are safe for employees, communities and consumers [22]. It is fair to say that the concept of sustainable manufacturing encourages innovation in production processes efficiency, thus adding more value to their products and services in favour of cleaner manufacturing [23]. The incentives for manufacturers to pursue sustainability have been researched in length [24]. It has been reported that those industries implementing sustainable practices not only can enhance quality but also attain a larger market share [25]. Moreover, manufacturing companies that have fundamentally changed their approach toward more sustainable manufacturing are proven to have consistent positive competitive results [26] despite the need to deliver increasingly customized products and services, with minimum cost and environmental impact [19].

According to a report published in 2021 by McKinsey Global Institute [27], manufacturing industries can get more positive outcomes on their invested capital by marketing their evolving value streams with minimum environmental impacts, and enhancing resource management such as recycling materials [19,28]. A more pressing matter is the drastic impact that climate change, induced by human actions, has on the world around us. The average temperature is expected to rise at least by 2.4 °C globally in less than a decade [29]. According to the report published by IPCC [30], to maintain the warming below 1.5 °C, we must eliminate carbon emissions by 2050 (Net Zero) and simultaneously reduce the emissions by 45% from 2010 to 2030 [31]. In order to deter further shifts in the environment with potentially unforeseeable consequences, many countries have announced greenhouse gas emission reduction plans for the upcoming decades [32]. Therefore, developing sustainable approaches for manufacturing companies has been regarded as a vital global affair [33]. Sustainability has been incorporated into manufacturing management aspects such as process optimisation [34], product development [35,36], supply chain management [37,38], lean manufacturing [39], and supplier evaluation and selection [40]. But none of them focuses exclusively on appraising environmental manufacturing performance [41].

It is clear that the most common view of sustainability stems from the TBL concept. The economic and social aspects are particularly developed, and although the environment is at the core of the sustainability dimension, it is deemed the most lacking aspect [42]. Therefore, the scope of this research is the reporting and analysing of the environmental sustainability of manufacturing systems. The literature reveals multiple attempts made by the researchers in approaching the subject of environmental assessment, such as the considerable body of work on accounting for carbon emissions [43] but it is generally limited to gas emissions based on the GHG protocol [44] or energy consumption [45]. Nevertheless, it should be noted that the aspect of environmental sustainability is concerned with the preservation of the natural basis of life and the security of the ecological conditions of human survival in general [21]. This represents the ability to maintain natural resources and preserve fundamental functions of the environment over time. Accordingly, considering gas emissions and energy consumption as the only negative impacts in terms of sustainability could be short-sighted [46], and more factors should be considered in any environmental sustainability analysis [47,48]. For instance, global material extraction has grown to more than three times what it was four decades ago while displaying no signals of deceleration and as a consequence, should be taken into account [49,50,51]. According to the review conducted by Contini and Peruzzini [52], the most widely used environmental indicator in manufacturing companies is “energy”, cited by 38 papers, out of 63. However, energy is not the only resource and gas emissions are not the only output. Various sets of key performance indicators (KPIs) are widely available that help define and quantify levels of sustainability in an organisation [53]. However, the recognition of KPIs merely does not allow for an evaluation of sustainability, unless the KPIs are prioritised with a clear assessment model [54].

This study aims to optimise sustainability reporting by developing a framework that allows for the consideration of all environmental indicators with influence on the manufacturing industry. This includes collecting, ranking, and composing a final KPIs set for specified targets. Consequently, the identified KPIs could be utilised as a part of sustainability strategies and projects or as standalone measures for reporting environmental manufacturing. Hence, the framework could aid both targeted evaluations and wider decision making. The next section presents the literature review conducted as part of the research. Section 3 presents the framework development followed by its validation in Section 4. Section 5 demonstrates the framework using a case study. Section 6 discusses the results and concludes the paper. Finally, Section 7 identifies limitations along with recommendations for future work.

## 2. Literature Review

The literature review sought to build on our understanding of sustainable manufacturing reporting methods and the accompanying factors considered. An examination of the available KPIs sets has been conducted as well as the different methods used to classify them.

### 2.1. Measuring and Reporting Sustainable Manufacturing Performance

It is fair to say that the concept of sustainable manufacturing encourages innovation in production systems efficiency leading to higher-value products in favour of cleaner manufacturing [23]. Therefore, it is evident that there is a need to measure the level of these sustainable practices in favour of economic advantages [55]. More importantly, industry and academia have reached a consensus that both a reduction of emission of greenhouse gasses and an overall promotion of sustainability hold the potential of mitigating or dampening the consequences of climate change. This led to various approaches for analysing and improving the sustainability of these business processes, but these approaches generally limit themselves to a few qualities which they assess (e.g., greenhouse gas emissions or energy consumption) and leave behind many others. Therefore, enterprises need clear methods and guidelines to support decision making by incorporating a system of measurement that address the sustainability expectations of both their internal and external stakeholders [12] and to design, operate, manage and finetune their manufacturing systems, aiming at environmental objectives to achieve the Eco-economy [19].

Sustainability strategies are the general description given to the methodology or agenda with the purpose of evaluating the impact of organisations’ actions. Such strategies are usually composed of a set of KPIs that define and quantify different aspects of an organisation [53]. Clearly defined KPIs are very useful in monitoring and enhancing manufacturing operations [56]. In light of reviewing the literature, implementation of frameworks has shown to be essential in maintaining company actions aligned with established strategic goals. Therefore, there is a need to investigate the development of such frameworks and select the right KPIs to facilitate the systematic evaluation of sustainability in order to enhance the decision-making process of the company. Indeed, a robust framework can help decision-makers overcome the challenges of corporate sustainability [57]. Especially if it allows them to better understand their current position and their desired end state. Despite several contributions, many corporations still find it challenging to cultivate and implement sustainability strategies [58].

Sustainability KPIs, indicators or metrics are all different terminologies given to the item of information collected to track the performance of an organisation or system at any operational level that produces output by consuming resources [19]. They form the building blocks of all sustainability strategies. Since the field of sustainability assessment is wide and complex, an increasing number of voluntary initiatives and companies have begun developing and using sustainability KPIs with minimal consideration given to the effect of selecting KPIs on their ability to orientate their decision-making process [19]. As a result, an abundance of metric lists and sets exists which generated confusion among manufacturers as they struggle to select an operational set of KPIs for evaluating sustainability in manufacturing. Specifically, manufacturing companies have been confronted with a decision on which KPIs to select for appraising their processes, and how they should interpret these KPIs in transforming their processes towards a sustainable future [59].

### 2.2. Sources of KPIs

As mentioned earlier, sustainability strategies have been vastly formulated and researched extensively. Subsequently, a great variety of KPIs related to sustainability in manufacturing has been developed, identified, specified, analysed and collected in published and restricted sources (patents and internal company documents). The literature review identifies two main sources of sustainability KPIs. The first source includes international bodies and organisations, such as Organisation for Economic Co-operation and Development (OECD) and Global Reporting Initiative (GRI) and many more as listed and reviewed in [60]. These organisations developed general and sector-specific KPIs. The major issue with using such KPIs is that they were developed with the purpose of reporting to external bodies. However, there is a lack of internal reporting guidance for manufacturing organisations and therefore, unsupportive of the decision-making process regarding localised environmental improvements. This revealed the need for a standardized framework for the sustainable manufacturing ecosystem. In this context, a standard methodology for identifying the right set of KPIs with clear definitions is still missing [61].

The second source consists of published research articles with different views and methods on choosing KPIs to include in sustainability strategies. These articles generally developed KPIs or collected and reviewed KPIs from other sources using a wide range of techniques and rationale. A noteworthy contribution can be found in [41] where an attempt was made to integrate sustainability into manufacturing performance by incorporating manufacturing performance KPIs with sustainability KPIs. As a result, a set of core metrics for sustainable manufacturing evaluation was suggested. However, renewable sources or alternative inputs were not considered as they do not directly affect manufacturing performance.

### 2.3. Sustainability KPIs Classification

A common category of KPIs is efficiency-based measurements, this includes several important KPIs that aim to assess the efficiency of a process or system based on information concerning the same resource or energy flow from two specific points of the system [43]. It allows its users to obtain a current outlook on the performance but does not necessarily identify specific areas of improvement. Fantini et al. [19] proposed a holistic framework for factories to assess their sustainability readiness by combining production efficiencies, economic performance and environmental impact with respect to time. The suggested framework, along with its developed KPIs, could be used for estimating the current position with respect to established sustainability targets with no possibility of further analysis.

In the same context, to mitigate the need for reviewing multiple sets of indicators, Hristov et al. [62] introduced composite KPIs which combine multiple aspects of system performance into a single measurement, based on a common scientific or economic standard. An example of these KPIs is ecological footprint (EF) which aimed to measure how much of the biosphere’s annual regenerative capacity is needed to renew the natural resource demand each year. Despite its ability to be used as a stand-alone tool for characterising over-consumption and its wider issues, research around its potential use suggests that the EF does not provide a meaningful demonstration of sustainability. Another common approach to identifying KPIs is to use the top-down and bottom-up perspectives. KPIs are developed from goals that are defined at a strategic level or specific targets set at an operational level of the production system. Nigri and Baldo [63] explored the use of such a method by developing a questionnaire based on the two perspectives and then ranking the KPIs based on expert feedback. However, the collection method of the KPIs is ambiguous and thus hard to follow. Mien et al. [36] also utilised organisational goals identified by conducting surveys where the most mentioned goals are picked. However, this method eliminated critical reasoning and relied purely on statistical data. The KPIs were then ranked by management for each goal but again, this can reject some KPIs that might be essential for a different situation, unless the entire method is performed repeatedly.

It seems that the incorporation of systematic sustainability in the organisation’s strategy is deficient as suggested by Yellishetty et al. [35]. Hence, some researchers suggested the use of a set of tools that are not necessarily purpose-built for sustainability but can act as a base or a supportive mechanism for the set of internally defined sustainability KPIs. For instance, industries can use process mapping, such as value stream mapping (VSM), not only for process assessment and development but also for identifying environmental improvement initiatives [63]. Such models can identify which points need to be improved along the production processes. However, because of the potential large number of manufacturing steps, the application of this methodology (VSM) in an entire company would be very time-consuming, particularly when introducing environmental aspects.

In summary, the literature review presented several KPIs utilised as a part of strategies of environmental analysis for the manufacturing industry. This led to the recognition of the following gaps which this paper will address: (1) Lack of a single set of KPIs that cover all aspects of environmental sustainability and (2) lack of a framework that incorporates all relevant environmental aspects into the manufacturing industries. This paper presents a structured framework for the manufacturing industries to identify the right environmental KPIs while addressing the aforementioned gaps.

## 3. Framework Development

**Figure 1:**
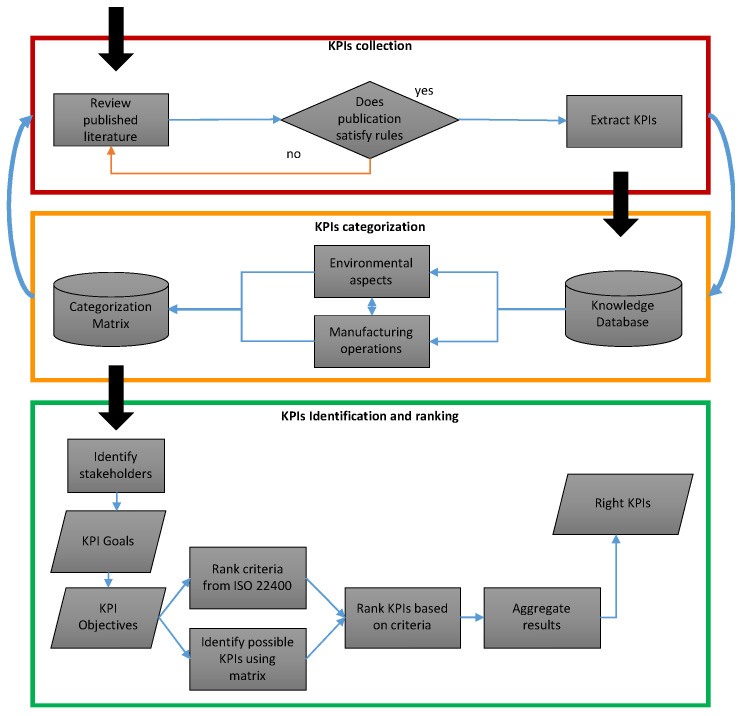
High-level schematic of the framework.

This section describes the development and utilisation of the proposed framework for environmental manufacturing assessment. It includes building a database for environmental KPIs, identifying, ranking, and composing a final KPIs set for specified targets. With continuous development in mind, further cultivation of the framework is essential for each industry to allow for the framework to better match its needs. The proposed approach will also allow for the incorporation of selection criteria that defines the usefulness of KPIs in the evaluation process rather than ranking the KPIs against each other. The framework comprises three stages as shown in Figure 1. The purpose of this framework is to provide a guide for the industry to build a database of potential KPIs and identify the right ones for specified objectives. The starting point involves collecting KPIs from available sources. Then the KPIs can be categorised based on environmental goals and manufacturing operations for easy identification. Finally, ranking and prioritising the right KPIs is performed based on a common set of selection criteria.

### 3.1. Collection of Initial KPIs: KPIs Knowledge Database

The collection exercise performed involves the accumulation of preliminary KPIs and selection criteria which are linked to the KPIs ranking method through a categorisation matrix. The more data is collected over time, the better the environmental analysis process. Sustainability is not a short-term approach to the development of the organisation. Hence, sustainability initiatives should be planned as both short-term and long-term actions to ensure that all stakeholder groups are satisfied [64]. Duflou et al. [65] noted that the selection of KPIs is necessary to assess the performance of a production management system. To build the initial knowledge database, the KPIs are selected from publications under specific conditions adapted from Murad [4]:

- Sufficient background research has been conducted prior to the method introduction
- The method should apply to the evaluation of the manufacturing sector
- The methodology should be in accordance with the TBL aspects
- The KPIs should be chosen or developed systematically with sufficient reasoning
- The evaluation method should apply to any stage or type of manufacturing processes

For the KPIs in specific literature to be selected, the publication should satisfy all the rules. Table 1 presents the list of articles selected for the KPIs collection procedure.

Being sustainable means managing processes and resources effectively from a long-term perspective [73]. Therefore, the KPIs collection procedure should be a continuous practice and should be constantly populated with new and up-to-date data by industry experts and managers leading to a database unique to each organisation. More specifically, they could search for KPIs without relying on rule E. As some KPIs sets can express industry-specific operations which could be an advantage to identify [74]. Sustainable performance and openness to the innovation of the industries are important requirements for such continuous development approach [75,76,77].

### 3.2. KPIs Categorisation

**Figure 2:**
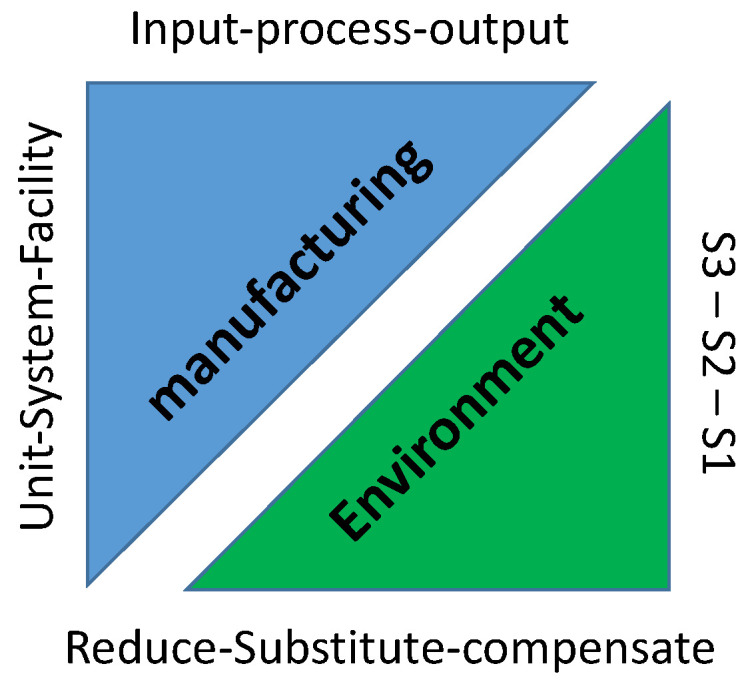
Categorisation outlook.

The size of the KPIs database would mainly depend on the organisation’s actions which leads to the possibility of having a large number of KPIs that deal with multiple aspects of manufacturing. However, selecting a small set of KPIs from many of those available for manufacturing operations is often not straightforward. Another issue is the identification of missed aspects that does not have a corresponding metric to analyse. This is mainly due to the complexity of synthesizing manufacturing operations and the environmental outlook. As seen from the literature review, despite the efforts made, synchronising the two may not be the easiest task. Therefore, this study tries to address this issue by elaborating on both elements (manufacturing and environment) individually and then combining them into a matrix which allows the categorisation of the collected KPIs. To allow for a full overview of the two elements, each element is divided into two dimensions, vertically and horizontally, as seen in Figure 2.

Firstly, the manufacturing element can be fairly viewed as an open system horizontally as it includes the exchange of energy and matter in and out of it with certain operations occurring in between. Here, a classification of the operations as input, processes and output was used to sort the collected KPIs [78]. On the other hand, achieving sustainability in manufacturing requires a holistic view spanning not just the product, and the manufacturing processes involved in its fabrication, but also the manufacturing system and the entire industry [60]. For that reason, it is most convenient when the considered appraisal for the process level could be expanded to provide a view at a higher level. Hence, the manufacturing operations were divided vertically as follows: unit process, multi-machine system, and facility [65].

Secondly, the environmental element was presented horizontally based on the institute of environmental management (IEMA) GHG management report [79]. It introduced a time-scaled plan of action to reduce, substitute and compensate for resource consumption and the impact of any organisational activities. It aims to help organisations recognise the urgency of their environmental impacts whenever decisions are being made on approaches to sustainable manufacturing. To put the matter in context:

- Reductions: KPIs that deal with real and relative factors with negative environmental impacts that require reduction, till elimination if possible. This includes the most pressing concern of carbon emissions as well as energy and resource management.
- Substitutions: KPIs that reflect on attributes with no further reduction possible but the negative impact could still be moderated by substituting aspects of the manufacturing operation. This includes the relatively untapped prospective of examining recycling and output reuse.
- Compensations: KPIs which represent any unavoidable or residual emissions, asset sharing, and carbon credits as well as considering actions beyond carbon neutrality.

**Figure A1:**
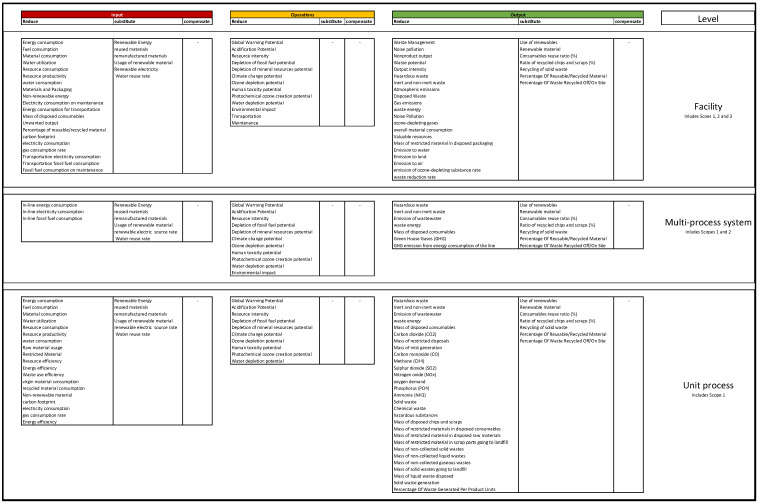
The collected metrics and the categorisation exercise performed as a part of the study to create initial knowledge database. Total number of metrics collected: 147, after removing duplicates: 111.

In terms of a vertical representation, the national grid’s scope 1, 2 and 3 of emissions and impact was chosen to represent the environmental KPIs. This is because it provides a necessary viewpoint on the direct impacts of the organisations’ actions, in addition to, indirect impacts, which are usually left unconsidered. More specifically, scope 1 deals with impacts from actions that an organisation initiates or controls directly. Scope 2 includes impacts that a company causes indirectly from the production of energy that they then purchase and use. Scope 3 incorporates impacts resulting from other organisations up and down the value chain [80]. In order to combine all the dimensions, a matrix was developed to map the collected KPIs as presented in Table 2. The collected KPIs and the categorisation exercise performed as a part of the study can be found in Figure A1 in the Appendix A.

### 3.3. Ranking of KPIs towards a Specific Goal

Table 2 proposed in the previous section could help in identifying KPIs relevant to the set targets. However, it is possible for a goal to have a few linked KPIs and therefore, it is important to determine which ones to prioritise. This section describes the method to systematically rank candidate KPIs in order of effectiveness towards achieving a specified target. It should allow for an understanding of how the KPIs compare to each other. The final score should represent the degree to which a metric satisfies a set of criteria. As each selection criterion is linked to a defined target, each metric is evaluated for its contribution to the target. Here, the steps involved are (1) identifying goals, (2) identifying stakeholders, (3) stakeholders selecting KPIs, (4) criteria are rated and then (5) KPIs are ranked.

The first step in the ranking of the KPIs is to determine the environmental goal to be reached. Such a goal could represent a generic aim or a target with a specific objective. In addition, the specific KPIs objectives may represent elements of the generalised goal. For the second step, stakeholders with direct ties to manufacturing operations need to be identified. Studies stress that there is value in involving the organisation’s main stakeholders in a standardised and authentic manner to become more sustainable, meaning that stakeholders should be at the base of the organisation’s strategy [81,82]. Accordingly, the proposed approach involves choosing multiple stakeholders to perform and develop the presented methods of further KPIs collection, categorisation and ranking. Stakeholders include line managers, supervisors, and shop floor workers who will perform the next three steps which represent the ranking calculations.

The ranking method suggested in this paper aims to evaluate the different KPIs collected and categorised. Kibira et al. [83] stated that the effectiveness of KPIs in representing a goal is assured by defining a set of selection criteria that the KPIs should follow. Nevertheless, not all the criteria are of equal influence on the KPIs and for that reason, the proposed method incorporates a set of criteria into the ranking procedure. They [81] suggest that the criteria should be gathered from the literature but this could lead to a discursive list. ISO 22400 offers a set of criteria that represent the usefulness of operational KPIs in a manufacturing setting along with their definitions. Eighteen criteria are extracted from the ISO document as listed in Table 3. Each criterion represents a characteristic that a KPI should be or have. Therefore, each one of the criteria is assigned a score ranging from 0 (not important) to 5 (very important) based on how important these attributes are to the KPIs in terms of dealing with the targets in question. This score is represented as C_i_. Stakeholders then select the KPIs corresponding to these targets using the knowledge database. The KPIs are then scored based on how well they satisfy each criterion on a scale of 0 (not satisfied) to 5 (completely satisfied), noted as K_i_. Finally, to produce a final score for each KPI, the score of each criterion, C_i_, is multiplied by the score of the KPI for this criterion, K_i_. this is expressed by:

(1) $$\mathrm{KPI}\;\mathrm{final}\;\mathrm{score}=\sum_{i=1}^{n}C_{i}\left(K_{i}\right)$$

where the KPIs with higher scores are considered more effective in representing the goal identified at the start. Subsequently, the stakeholders can determine a cut-off point for the scores and only take into account the consequent KPIs.

This methodology agrees with the contingent theory that advocates the unavailability of a single right answer [84] but identifying the optimum course of action is dependent upon the circumstances of each organisation. Following such theory, it allows the method to be both systematic in nature yet agile enough to be utilised in many situations.

## 4. Framework Validation

**Figure 3:**
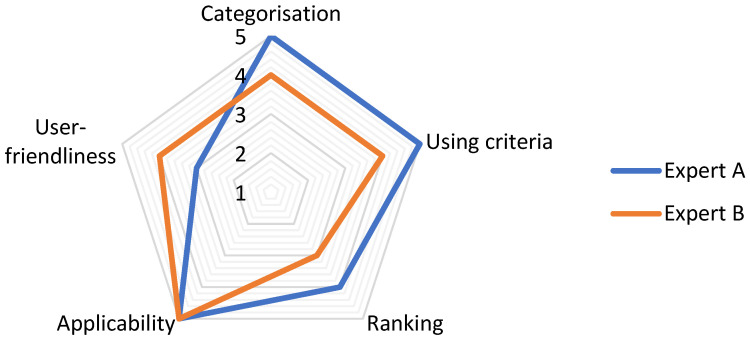
Validation results.

To validate the developed framework, two sustainable manufacturing experts were presented with the steps involved in collecting and categorising environmental KPIs, together with the procedure to rank indicators based on their effectiveness in representing a specified target. Using the following set of statements, the developed framework was quantitatively assessed by rating each statement on a five-point Likert scale ranging from 1 (strongly disagree) to 5 (strongly agree):

- Categorisation successfully integrates environmental sustainability in manufacturing operations
- Criteria aid in choosing KPIs by representing their usefulness/effectiveness
- Ranking results would support decision making by prioritising KPIs
- The framework has potential applicability in manufacturing organisations
- The approach is user-friendly

where the first three statements are specific to the elements of the framework while the last two represent a more general overview of the applicability and usage of the framework. The results are plotted in Figure 3.

Overall, the results indicate a positive stance on the developed framework. More specifically, the potential applicability of the framework has been corroborated by both experts. Moreover, the continuous approach for collecting and categorising KPIs to allow for creating a unique database for each organisation has been acknowledged positively. However, minor remarks were made on the user-friendliness of the ranking method as it is not instantly intuitive but requires a thorough explanation.

## 5. Framework Demonstration

Using a manufacturing case study, this section investigates the viability of the procedure described earlier for identifying the right KPIs. This includes analysing the knowledge database built as a part of this research as well as testing the proposed ranking method. To accomplish this, informal interviews were conducted with experts from two glass industry companies, denoted as companies A and B. First, open questions were asked to grasp their current environmental assessment methods and any indicator sets they utilise. Second, their input was acquired on the framework by presenting the different steps involved. Additionally, a case study was performed for the ranking process using a target the interviewee chooses. This allowed for identifying possible benefits and limitations. For the ranking stage, the interviewees were asked to select a goal from the following options which were based on operational goals from several manufacturing industries:

- Reduce energy use
- Zero waste to landfill
- Reduce gas emissions
- Increase renewable electricity

Then, they assigned an importance level for some criteria they deemed immediately crucial. The next step was for them to identify possible KPIs using the Knowledge database and rank them using the method described in Section 3.3.

### Case Study

Company A indicated that they rely on qualitative methods that do not incorporate KPIs while deciding on environmental sustainability actions. On the other hand, company B stated that KPIs are a big part of their environmental plans based on the TBL paradigm with a focus on legal compliance. However, they are usually developed for specific projects and only cover individual aspects concerning the project with no procedure in place to select, record or classify them. Accordingly, they both expressed appreciation for building a database with a categorisation method that focuses on manufacturing operations.

To perform the ranking stage, company A selected reducing energy at the process level as their target while company B selected increasing renewable electricity at a facility level. Table 4 and Table 5 present the ratings assigned by the interviewees of companies A and B, respectively, for the selected KPIs for each criterion and the calculated final score of each KPI.

For company A, the results revealed that the KPIs are ranked as follows: (1) electricity consumption, (2) fossil fuel consumption, (3) fuel consumption, (4) energy efficiency and (5) energy waste. The industry expert then elected 40 as the cut-off point and identified the top 3 KPIs to be the right ones for measuring the target out of the selected KPIs. For company B, only two KPIs were found relevant to the goal which implies a lack of KPIs aimed at renewable energy management in the database.

## 6. Discussion and Conclusions

In this paper, a framework of environmental KPIs selection for manufacturing industries has been presented. Numerous studies stressed the significance of implementing eco-friendly activities on both profitability and assisting in the mitigation of irreversible environmental circumstances. Nevertheless, several sustainability assessment methods that feature KPIs have been reported in the literature with minimal emphasis on covering all manufacturing aspects that impact the environment.

The work started by collecting KPIs from published articles that satisfy a set of rules to ensure their appropriateness. The collected indicators were then categorised based on the environmental aspects and the manufacturing operations. To discover the usefulness of the different KPIs, a ranking method was introduced that includes selection criteria in the final score of each KPI. Then, using a quantitative survey, the framework was validated by subject matter experts. Finally, the viability of the framework has been investigated using a manufacturing case study to practically ascertain its benefits and reveal any limitations. The framework was accepted by both subject matter and industry experts as means to categorise KPIs and evaluate their effectiveness. More specifically, the categorisation method showed its capability to embrace aspects of both manufacturing operations and environmental sustainability. Additionally, it aided in choosing KPIs and in identifying areas that lack monitoring. Incorporating selection criteria assisted in identifying the most effective KPIs for a specified goal. It was pointed out by one of the participants that the ranking process is particularly helpful with identifying the right KPIs between a list of indicators with similar emphasis as well as supporting decision-making by prioritising KPIs.

## 7. Limitations and Future Research

While the framework has been validated as a step towards enabling manufacturing industries to build, embed and sustain a strategic organisational approach to environmental manufacturing, it is limited in some aspects. Theoretically, the method is limited by the research available on the subject which constrains the possible number of KPIs that could be collected and categorised. Furthermore, the method is limited practically by the collection exercise that should be performed as part of the framework. In the case of the database built as part of the research, it is limited to the genericness of the initially collected indicators. Both companies involved in the case study have stated their interest in using the categorisation method with a collection of industry-specific KPIs. In the case of the glass industry, this could include indicators such as cullet utilisation and reuse of collected dust [85,86]. This leads to the next research step which involves further building the knowledge database with the aim of collecting KPIs that fulfil the underacknowledged categorisation sections. For instance, operations substitutions could include indicators representing the introduction of environmentally positive processes to an existing system such as biomethanisation and carbon capture as suggested by one of the industry experts. Further work might aim to investigate the utilisation of the identified KPIs with an indication of how to achieve specified targets by using those KPIs.

## Figures and Tables

**Table 1:** Result of analysis of publications presenting sets of sustainability KPIs.

	Title	Rules					Selected
		A	B	C	D	E	
[60]	Key Performance Indicators for Sustainable Manufacturing Evaluation in Industry	y	y	y	y	y	√
[66]	Proposed Framework for Assessing the Sustainability of Membrane Life Cycle	y	y	y	y	y	√
[19]	Back to Intuition: Proposal for a Performance Indicators Framework to Facilitate Eco-factories Management and Benchmarking	y	y	y	y	y	√
[67]	Review of Existing Sustainability Assessment Methods for Malaysian Palm Oil Production	y	y			y	x
[68]	Selection Criteria for Suitable Indicators for Value Creation Starting with a Look at the Environmental Dimension	y	y	y	y	y	√
[4]	Metric-based approach to assess sustainable manufacturing performance at manufacturing process levels	y	y	y	y	y	√
[69]	How Soft Drink Supply Chains drive sustainability: Key Performance Indicators (KPIs) identification	y	y	y	y	y	√
[62]	The adoption of the key performance indicators to integrate sustainability in the business strategy: A five-dimensional framework	y	y				x
[70]	Environmental KPI Selection Using Criteria Value and Demonstration	y		y			x
[42]	The Role of Sustainability Key Performance Indicators (KPIs) in Implementing Sustainable Strategies	y	y	y	y	y	√
[59]	Categorization of indicators for sustainable manufacturing	y	y	y	y	y	√
[71]	Sustainable Value Stream Mapping (Sus-VSM): methodology to visualize and assess manufacturing sustainability performance	y	y	y	y	y	√
[72]	A Metrics-based Sustainability Assessment of Cryogenic Machining Using Modelling and Optimization of Process Performance	y	y	y	y	y	√

**Table 2:** KPIs categorisation overview (R: reduction, S: substitution, C: compensation, s: scope).

	Manufacturing → ↓ Environment	Input			Operation			Output			Scope
		R	S	C	R	S	C	R	S	C	
**Operation Levels**	Facility										s1, s2 and s3
	Multi-process system										s1, s2
	Unit process										s1

**Table 3:** Criteria extracted from ISO 22400.

Accurate	Automated	Complete
Actionable	Buy-in	Documented
Aligned	Comparable	Inexpensive
Timely	Understandable	Standardized
Trackable	Valid	Unambiguous
Predictive	Quantifiable	Relevant

**Table 4:** Case study result of company A—target: reduce energy use.

		KPIs				
	Criteria	Energy Efficiency	Fuel Consumption	Electricity Consumption	Energy Waste	Fossil Fuel Consumption
4	Accurate	4	4	5	3	5
3	Trackable	2	4	5	2	4
2	Quantifiable	4	5	5	5	5
1	Inexpensive	3	3	5	4	4
	Final Score	33	41	50	32	46

**Table 5:** Case study result of company B—target: increase renewable electricity.

		KPIs	
	Criteria	Renewable Energy	Renewable Electricity
4	Trackable	5	5
3	Inexpensive	2	2
2	Timely	3	3
1	Quantifiable	4	5
	Final Score	36	37

## Data Availability

Data is contained within the article.
